# Cytoadherence phenotype of *Plasmodium falciparum-*infected erythrocytes is associated with specific *pfemp-1* expression in parasites from children with cerebral malaria

**DOI:** 10.1186/1475-2875-13-333

**Published:** 2014-08-25

**Authors:** Talleh Almelli, Nicaise T Ndam, Sem Ezimegnon, Maroufou J Alao, Charles Ahouansou, Gratien Sagbo, Annick Amoussou, Philippe Deloron, Rachida Tahar

**Affiliations:** Institut de Recherche pour le Développement (IRD), UMR 216 Mère et Enfant Face aux Infections Tropicales, 4, avenue de l’Observatoire, Paris, 75270 France; PRES Sorbonne Paris Cité, Faculté de Pharmacie, Université Paris Descartes, Paris, France; Centre d’Etude et de recherche sur le Paludisme Associé à la Grossesse et l’Enfance (CERPAGE), Cotonou, Bénin; Département de pédiatrie, Hôpital Mère-enfant de la lagune (HOMEL), Cotonou, Bénin; Service de pédiatrie, Centre National Hospitalo-Universitaire (CNHU), Cotonou, Bénin; Service de pédiatrie, Hôpital de zone de Suru-Léré, Cotonou, Bénin

**Keywords:** *Plasmodium falciparum*, Cerebral malaria, Uncomplicated malaria, *Var* genes, Domain cassette, Transcript abundance, Cytoadherence, CD36, ICAM-1, CSPG, CSA

## Abstract

**Background:**

Cytoadherence of *Plasmodium falciparum-*infected erythrocytes (IEs) in deep microvasculature endothelia plays a major role in the pathogenesis of cerebral malaria (CM). This biological process is thought to be mediated by *P. falciparum* erythrocyte membrane protein-1 (PfEMP-1) and human receptors such as CD36 and ICAM-1. The relationship between the expression of PfEMP-1 variants and cytoadherence phenotype in the pathology of malaria is not well established.

**Methods:**

Cytoadherence phenotypes of IEs to CD36, ICAM-1, CSPG and the transcription patterns of A, B, var2csa*, var3, var* gene groups and domain cassettes DC8 and DC13 were assessed in parasites from children with CM and uncomplicated malaria (UM) to determine if cytoadherence is related to a specific transcription profile of *pfemp*-1 variants.

**Results:**

Parasites from CM patients bind significantly more to CD36 than those from UM patients, but no difference was observed in their binding ability to ICAM-1 and CSPG. CM isolates highly transcribed groups A, B, var2csa*, var3*, DC8 and DC13 compared to UM parasites. The high transcription levels of *var* genes belonging to group B positively correlated with increased binding level to CD36.

**Conclusion:**

CM isolates bind significantly more to CD36 than to ICAM-1, which was correlated with high transcription level of group B *var* genes, supporting their implication in malaria pathogenesis.

## Background

Cerebral malaria (CM) is one of the serious manifestations of *falciparum* malaria. Despite an adequate treatment, 15-20% of infants presenting with CM die in hospital [1]. There is strong evidence that sequestration of *Plasmodium falciparum*-infected erythrocytes (IEs) within the brain microvascular endothelia plays a key role in CM pathogenesis [2, 3]. This and comparable in CM and UM isolates. Howeverphenomenon protects the mature parasites from spleen-dependent clearance [4]. The adhesive properties of IEs are attributed to *P. falciparum* Erythrocyte Membrane Protein-1 (PfEMP-1) expressed at the surface of IEs [5]. Encoded by ~60 members of *var* gene family, *pfemp-1* genes are divided into three main groups (A, B and C) and three relatively conserved groups *var1,* var2csa and *var3* [6]. It has been shown that groups A and B *var* genes are differentially transcribed in isolates associated with severe malaria, compared with parasites from uncomplicated malaria (UM) or asymptomatic infections [7–9]. Recent studies have shown that PfEMP-1 s are characterized by 23 conserved architectural motifs named domain cassettes (DC) and that DC8 and DC13 are of clinical significance. The transcription levels of these DCs are higher in parasites from severe malaria, including CM, than in those from UM [10, 11]. Moreover, *P. falciparum* parasites selected for binding to human brain microvascular endothelial cells (HBMEC) express high levels PfEMP-1 DC8 genes [12], and plasma from convalescent patients exhibited higher antibody recognition profiles to HBMEC-selected parasites than unselected ones, suggesting the importance of PfEMP-1 motifs in building protective immunity to malaria [12, 13].

*In vitro* experiments identified a few endothelial receptors that participate in the adherence of IEs to microvasculature endothelia. CD36 and Inter Cellular Adhesion Molecule-1 (ICAM-1) are the most commonly used receptors by clinical isolates [14]. As yet, apart from chondroitin sulphate A proteoglycan (CSPG), which is the main receptor of parasites isolated from pregnant women, no definite candidate has been determined for cerebral malaria pathology [15]. It has been shown that parasites infecting children or non-pregnant host have very low binding to CSA, suggesting a role of other receptors in the pathology of malaria in non pregnant women [16, 17]. CD36 is considered as a common receptor involved in the cytoadherence of *P. falciparum* isolates [18–20]. Several studies have found no difference in CD36-binding patterns between parasites from severe malaria and those from UM patients [17, 21], while others have reported an association between UM and high binding level to CD36 [22, 23]. Post-mortem brain examination has reported high levels of ICAM-1, which co-localized with IEs in cerebral blood vessels [24]. This finding is in agreement with studies that suggested an association between disease severity and ICAM-1-binding [23]. However, some studies did not detect any difference in binding to ICAM-1 in parasite isolates from severe or mild cases [20, 25].

The involvement of CD36 and ICAM-1 as well as that of PfEMP-1 variant expression in the pathogenesis of CM has not been well established. Recently, Turner *et al* identified the Endothelial Protein C Receptor (EPCR) as a new mediator of IEs adhesion to microvascular endothelial cells via the interaction with domain cassette 8 and 13 of *PfEMP-1* variants.

To investigate the relationship between PfEMP-1 variant expression and cytoadhesion to CD36, ICAM-1 and CSPG the binding phenotype as well as the transcript abundance of pfemp-1 variants of parasites freshly collected from Beninese children with CM or UM was carried out by static binding assay and rt-qPCR respectively. The binding assay to EPCR was not investigated in this study.

## Methods

### Ethics statement

This study was reviewed and approved by the ethics committee of the Research Institute of Applied Biomedical Sciences, Cotonou, Benin (No 006/CER/ISBA/12 and No 21/CER/ISBA/13).

### Study design, malaria patients

This study was conducted in Cotonou, southern Benin, during the 2012 and 2013 malaria transmission periods (June-September and May-July, respectively). This area is characterized by two rainy seasons during which malaria infection is mainly caused by *P. falciparum*, with approximately 33 infective bites per person annually [26].

Children under five years of age spontaneously presenting at Hôpital Mère-enfant de la Lagune (HOMEL), Centre National Hospitalier Universitaire Hubert Koutoucou Mega (CNHU-HKM), or to Hôpital Suru-Léré were screened by rapid diagnostic test for malaria (DiaQuick Malaria *P. falciparum* Cassette, Dialab®; Hondastrasse, Austria) and were recruited in the study if they presented CM or UM. CM was defined as a microscopically confirmed *P. falciparum* infection and a Blantyre coma score ≤2, with the exclusion of any other causes of coma. UM was defined as *P. falciparum* parasitaemia accompanied by fever, headache, or myalgia without signs of severity and/or evidence of vital organ dysfunction, as defined by the World Health Organization (WHO) [27]. After obtaining informed and written consent from parents or guardians, 2 to 4 ml of venous blood samples were collected into tubes containing citrate phosphate dextrose adenine. *Plasmodium falciparum* infections were confirmed by microscopic examination of Giemsa-stained thick blood smears, and parasitaemia was recorded as the number of asexual parasites/μL of blood. All participants were treated according to the guidelines established by the Beninese Ministry of Health.

### Sample preparation and parasite culture

Plasma and buffy coat were removed from fresh parasitized blood samples, and red cell pellet was washed twice in RPMI 1640 medium (LONZA, Amboise, France) containing 50 mg/ml gentamicin by centrifugation (800 *g* × 10 min). One-hundred μl were conserved in TRIzol® reagent (Life Technologies, Illkirch, France), and stored at -80°C for RNA extraction. Another 100 μl were spotted on Whatman 3MM filter paper, and stored at room temperature for DNA extraction and merozoite surface protein-1 (*msp-1*) and *msp-2* genotyping. Five-hundred μl were cultured in RPMI 1640 medium supplemented with 10% human serum. Parasites were cultured for less than 48 hours until they reached the mature stage (mature trophozoites and schizonts) and purified using magnetic columns (MACS, Milteny Biotec, Bergisch Gladbach, Germany) for binding assay.

### Binding assay

Static binding assays were performed with 27 UM and 38 CM fresh isolates at 20% parasitaemia in 100 × 15-mm Petri dishes as described [28]. Twenty μl of each recombinant protein diluted in phosphate buffer saline (PBS) were spotted on Petri dish and incubated overnight at 4°C in a sealed humid container. The following recombinant proteins were used: CSPG at 5 μg/ml (Decorin, Sigma-Aldrich, Saint-Quentin Fallavier, France), CD36 and ICAM-I at 10 μg/ml (R&D, Lille, France). The spots were blocked with 3% bovine serum albumin (BSA) in PBS for 30 min at 37°C in a humid chamber. Purified parasites were washed with PBS, centrifuged for 10 min at 800 *g*, resuspended in 3% BSA in RPMI 1640 medium and 20 μl of parasite suspension were added to each spot and incubated for 12 min at room temperature. Unbound erythrocytes were washed off gently with PBS using an automated washing system. Bound cells were fixed with 1.5% glutaraldehyde in PBS for 10 min, Giemsa-stained and counted microscopically. The number of IEs bound to each receptor was determined by counting ten fields using a 40× objective, and expressed as the number of IEs bound per sq mm. For each sample, the binding assay was done in duplicate for all proteins in the same plate. Results were expressed as the mean binding level of duplicate spots per sample. As a negative control, 1% BSA was used to assess non-specific binding. This protocol has been standardized using FCR3-CSA and FCR3-CD36 binding parasite strains.

As documented in several studies CSA binding phenotype is highly correlated with the expression of VAR2CSA in IEs from pregnant women [29–31]. Therefore, CSA binding experiment and *var2csa* transcript abundance assessment as a second negative control were included. The binding to CSA and transcription of *var2csa* in parasite from children are expected to be low or undetectable.

### Parasite RNA extraction and cDNA synthesis

cDNAs were synthesized from 36 UM and 51 CM samples preserved in TRIzol® reagent, following the manufacturer’s instructions. RNAs were treated with DNAse I (BioLabs, Ipswich, MA), and the absence of genomic DNA was assessed by 40 cycles of RT-PCR with *fructose-biphosphate aldolase* primers [29]. Reverse transcription of RNA was performed by Thermoscript® (Life Technologies) with random hexamers and oligo dt primers, following manufacturer’s recommendations.

### Transcript abundance level of *var*genes

Individual RT-PCR reactions were performed using Rotorgene® thermal cycler (Corbett Research) with 1X SYBR Green® (Bioline) and 1.25 μM of specific primer pairs for group A (A1, A2, A3), group B (B1), *var2csa* and *var3* of *var* genes. A set of primers that target DC8 and DC13 was also used [10]. The endogenous controls used were *fructose-bisphosphate aldolase* and *seryl-tRNA synthetase* [29]. Cycling conditions were 95°C for 1 min, followed by 40 cycles of 95°C for 30 sec, 54°C for 40 sec, and 68°C for 50 sec. Data were analysed by the Rotorgene software for which the threshold cycle was set at 0.025. The specificity of each PCR product was verified by the specific melting curve for each pair of primers. The transcript abundance was calculated by relative quantification using two housekeeping genes (∆Ct _var_primer_ = Ct _vr_primer_ - Ct _average_control primers_). Transcript units were determined as Tu = 2^(5-ΔCt)^ [10].

### Genomic DNA extraction and *msp1*and *msp*2 genotyping

Genomic DNA was extracted from filter papers using the Chelex 100 resin method [32]. Specific primers were used to amplify block 2 of *msp-1* and block 3 of *msp-2* [33]. Multiplicity of infection (MOI) was defined as the highest *msp-1* or *msp-2* allele number found in each sample.

### Statistical analysis

Binding data were expressed as the number of IEs/mm^2^ bound to a given receptor minus the number of IEs bound to BSA. The non-parametric Mann-Whitney U test was used to compare the binding levels to receptors and the transcript abundance of *var* group genes and DC8 and DC13 between CM and UM groups. Correlation coefficient between binding profile and transcript level of dominant *var* group, which was transcribed in more than 50% of samples, was calculated by the Spearman’s rank test. Data were plotted and statistical tests were performed using Prism v5 software (GraphPad Software, Inc., San Diego, CA, USA). The level of statistical significance was set at 0.05.

## Results

A total of 88 children were enrolled, including 52 children with CM and 36 with UM. Of 52 children with CM, 21 presented CM alone, while the others had overlapping clinical syndromes: 25 with severe anaemia (SA), and 16 with hyperparasitaemia (HP) defined as parasite density ≥250.000 p/μl (ten children with CM presented both SA and HP). The clinical and biological features of these patients are summarized in Table 1. There was no difference (*P* >0.05) in the mean age, sex ratios and mean body temperature between the two groups. However, the mean haemoglobin level was lower (P <0.0001), while the mean parasite density was higher (P <0.01) in CM group than UM group. All children with UM survived, while 19 patients (36.5%) with CM died. Multiple infections were recorded in both groups, with a mean MOI of 3.4 distinct parasite populations in CM and 2.2 parasite populations in UM group (P <0.0001), These MOI values are consistent with the high endemicity of malaria in the study site.

**Table 1:** **Parasitological and clinical characteristics of children enrolled in Cotonou, 2012-2013**

	Cerebral malaria (N = 52)	Uncomplicated malaria (N = 36)	P-value
Sex ratio (female/male)	23/29	18/18	0.2
Age (months), median [range]	42 [5-72]	33 [8-58]	0.09
Parasitaemia (P/μl), median [range]	75,806 [889-3,600,000]	49,453.5 [1,164-246,857]	0.01
Haemoglobin (g/dl), median [range]	5.15 [0.8-12.6]	7.45 [5.2-12.5]	0.0001
Blantyre score, median [range]	2 [0-2]	-	-
Temperature, median [range]	38°C [35.5-40°C]	38.5°C [36.5-40°C]	0.15
Multiplicity of infection, median [range]	3.4 [1-6]	2.2 [1-6]	0.0001
Number of deaths	19	0	-

### Binding profiles

Due to the low parasitaemia of some UM isolates (geometric mean parasite density, 23,078 asexual parasites/μl range, (75-246,857) asexual parasites/μl) and anti-malarial treatment before enrolment in some of the CM children, *in vitro* parasite cultivation was successful among 27 of 36 isolates in the UM group and 38 of 52 isolates in the CM group. The binding phenotypes were analysed in 65 isolates that developed into mature forms. All but two isolates (63 of 65) bound to both CD36 and ICAM-1, and 48 isolates bound weakly to CSPG (Table 2).

**Table 2:** **Receptor binding data**

	Cerebral malaria	Uncomplicated malaria	All samples
	(n = 38)	(n = 27)	(n = 65)
CD36	350.5 (0-2552)	85.1 (0-379)	244.0 (0-2552)
ICAM-1	48.3 (0.6-215)	39.3 (0-294)	45.2 (0-294)
CSPG	11.3 (0-62)	9.6 (0-72)	10.7 (0-72)

Values are the mean number of infected erythrocytes bound (range)/mm^2^ to a given receptor minus the number of infected erythrocytes bound to BSA.

**Figure 1:**
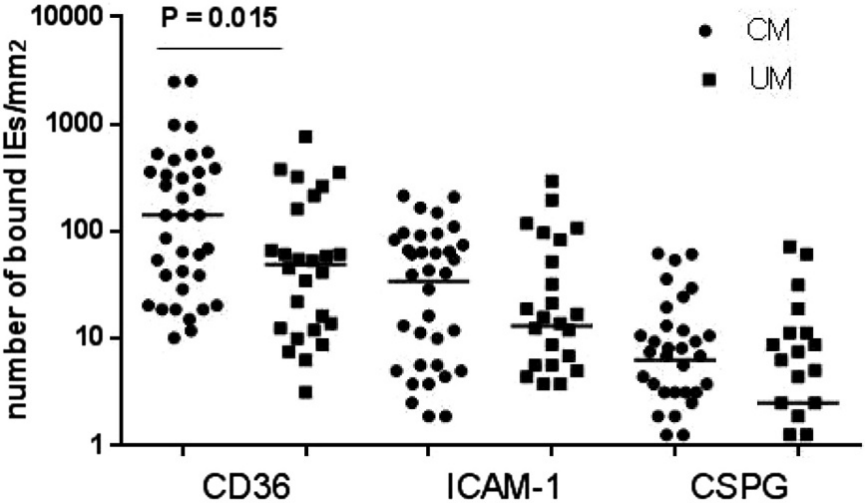
**Binding level (number of bound IEs/mm** ^**2**^ **) of CM and UM isolates on CSPG, ICAM-1 and CD36.** The difference in binding levels to a specific receptor was tested by the Mann-Whitney U test, significant *P*-value is shown in the figure.

Cytoadherence levels were compared between CM and UM samples separately to each receptor. The binding of CM isolates to CD36 was significantly higher (*P* = 0.015) than that of UM samples. However, there was no significant difference in the binding level to ICAM-1 (*P* = 0.4) or to CSPG (*P* = 0.1) between CM and UM isolates Figure 1.

### Transcript levels of *var*gene groups, DC8, and DC13

**Figure 2:**
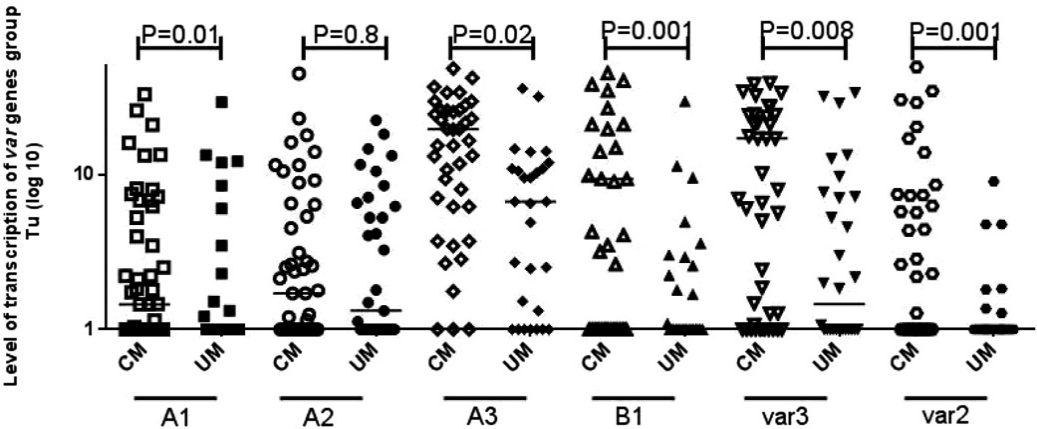
**Transcript abundances of** ***var*** **genes from group A, B,** ***var2csa*** **and** ***var3*** **in** ***Plasmodium falciparum*** **isolates from children presenting with either cerebral (CM) or uncomplicated malaria (UM).** Transcript patterns of specific *var* genes were compared between groups by the Mann-Whitney U test, significant *P*-values are shown in the figure.

**Figure 3:**
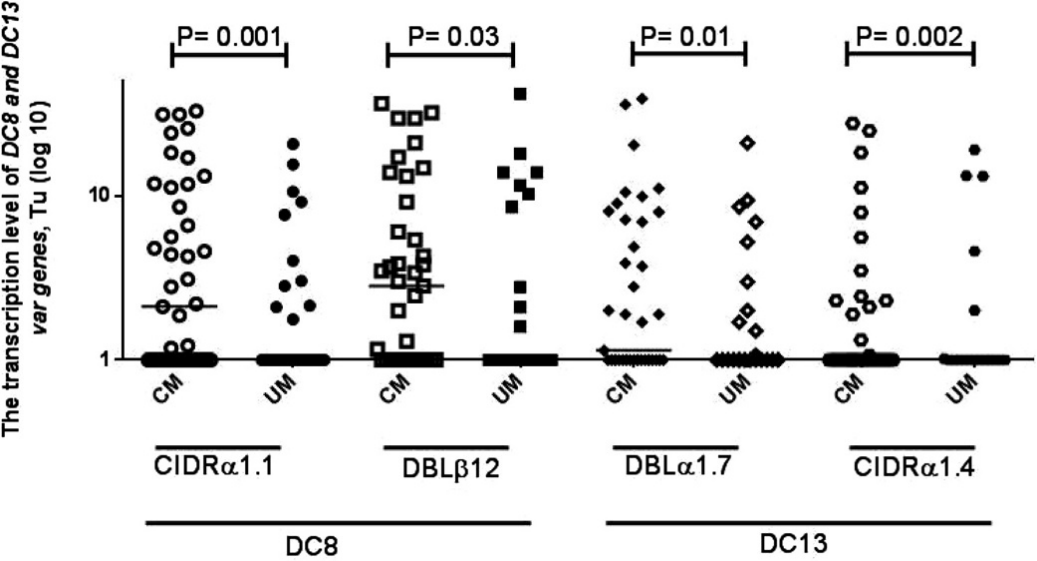
**Transcript abundances of DC8 and DC13 targeted by CIDRα1.1, DBLβ12, DBLα1.7, and CIDRα1.4 primers in** ***Plasmodium falciparum*** **isolates from children presenting with either cerebral (CM) or uncomplicated malaria (UM).** Transcript patterns were compared between groups by the Mann-Whitney U test, significant *P*-values are shown in the figure.

The transcription level of A, B,*var2csa, var3*, DC8, and DC13 *var* genes was analysed by RT-qPCR in 36 UM and 51 CM isolates. Group A, B, *var2csa, var3,* DC8, and DC13 had significantly higher levels of transcription (P <0.05) in CM isolates than in UM isolates (Table 3, Figures 2 and 3). However, no difference in the transcription level was observed between CM and UM isolates when genes were targeted by A2, DBLγ4/6 and DBLαCIDRα primer sets.

**Table 3:** **Transcript abundances of** ***var*** **genes from groups A, B, var2csa** ***, var3*** **, DC8 and DC13**

Primers ID	T _u__CM median [IQR]	T _u__UM median [IQR]	***P***-value
A1 (n = xx)	1.4 [1-6.8]	1 [0-1.7]	0.01
A2 (n = xx)	1.2 [1-5.3]	1 [1-6.3]	0.8
A3 (n = xx)	19.6 [4.3-33]	6.6 [1-14.3]	0.02
B1 (n = xx)	9.3 [1-64]	1 [1-2.6]	0.001
var2 (n = xx)	1 [1-4.4]	1 [0-1]	0.001
var3 (n = xx)	17.1 [1-34]	1.4 [1-10.4]	0.008
CIDRα1.1 (DC8) (n = xx)	2.11 [1-11.8]	1 [1-2.3]	0.02
DBLβ12 (DC8) (n = xx)	2.8 [1-13.9]	1 [1-4.2]	0.03
DBLγ4/6 (DC8) (n = xx)	1 [0-1]	1 [0-1]	0.4
DBLαCIDRα (DC8) (n = xx)	0 [0-1]	0 [0-1 ]	0.4
DBLα1.7 (DC13) (n = xx)	1.1 [1-8.1]	1 [ 0-1.5]	0.01
CIDRα1.4 (DC13) (n = xx)	1 [1-2.2]	1 [ 0-1]	0.002

Comparisons between *P. falciparum* isolates from children presenting with cerebral (CM) and uncomplicated malaria (UM) were achieved by the Mann-Whitney U test.

### Correlation between binding phenotype and transcription level of *var*genes

The relationship between binding profiles and transcription level of *var* gene groups and DCs for each isolate was examined by considering only highly transcribed genes that were determined as ‘predominant transcripts’.

Group B *var* genes were the predominant transcripts in 51% of samples. Tu value of group B *var* genes was positively correlated with the binding level to CD36 (r = 0.46, *P* = 0.005), but not with binding to ICAM-1 (r = 0.05, *P* = 0.7) or CSPG (r = 0.1, *P* = 0.2). Group A *var* genes were the predominant transcripts in 49% of samples, and no significant correlation was observed between Tu values and binding levels to CD36 (r = 0.2, *P* = 0.2), ICAM-1 (r = 0.1, *P* = 0.4), or CSPG (r = -0.02, *P* = 0.1).

Likewise, DC8 and DC13 genes were predominant in 78.5 and 21.5% of samples, respectively. Tu values of DC8 and DC13 were not correlated with binding the levels to CD36(r = -0.005, [P = 0.9] and r = 0.09 [P = 0.7], respectively), ICAM-1 (r = 0.1, [P = 0.4] and r = -0.06, [P = 0.8], respectively) and CSPG (r = 0.05 [P = 0.7] and r = -0.1 [P = 0.5], respectively).

## Discussion

Cytoadherence of IEs to cerebral microvasculature cell lining is considered to be the major factor in CM pathogenesis [2, 34]. This mechanism is known to be mediated by PfEMP-1 variants expressed at the surface of IEs [35]. However, the exact ligand-receptor interactions leading to the binding of IEs to brain microvascular endothelium are not well understood.

To investigate the potential role of PfEMP-1 variants in cytoadherence to host receptor, clinical isolates from patients with cerebral or uncomplicated malaria were used to assess the cytoadherence phenotypes to ICAM-1, CD36, and CSPG and the transcription profiles of *var* genes.

The results suggest that CD36 is a common binding receptor used by clinical isolates from both UM and CM, in agreement with earlier findings that showed the involvement of CD36 in cytoadherence [14, 18, 19]. However, contrary to the results of previous studies, CD36-binding level was shown to be higher in isolates from CM group than those from UM group, suggesting the key role played by CD36 in parasite sequestration during CM. This result oppose to the likely admitted assumption considering CD36 binding phenotype as common phenotype unrelated to parasite virulence.

Conversely, in agreement with previous studies using static binding assay [20, 25], the binding level to ICAM-1 was low and comparable in CM and UM isolates. However, a higher binding level to ICAM-1 in CM isolates, as compared to that of UM isolates, was seen under flow conditions, as probably flow assay better simulates physiological wall shear stress under which ICAM-1 promotes IEs rolling and binding [23]. Hence, the lower level of parasite binding to ICAM-1, as compared to CD36, observed in this study may also occur due to suboptimal interaction between ICAM-1 and IEs resulting from conformational changes of this receptor when immobilized on a plastic surface. Moreover, the significantly higher MOI in CM parasites may also endow CM group with higher number of parasites with CD36 binding phenotype.

The development of new binding assays closely mimicking physiological ligand-receptor interaction will provide relevant knowledge on receptor affinity to IEs. Moreover, it has been shown that *P. falciparum* isolates, including those from CM, bind to CD36 ten-fold higher than to ICAM-1, and that 80% of ICAM-1-selected IEs bind to CD36, suggesting an association of CD36 with disease pathogenesis as it facilitates the binding of a high proportion of parasites to the endothelium in various deep organs [36]. In addition, under flow conditions, CD36 offers greater and more efficient adherence property than ICAM-1 [37]. These results are in agreement with data of this study showing a higher binding level of IEs to CD36 than ICAM-1.

Despite the fact that CD36 is poorly expressed in brain vascular endothelia, Turner *et al.* reported low but significant association between CD36 expression and IEs sequestration in brain vessels [38]. Moreover, IEs binding to cerebral endothelial cells may take place indirectly through platelets accumulated in the brain vasculature of CM patients [39]. Platelets express a high level of CD36, which may promote IEs binding and sequestration in the brain, despite the low CD36 expression on brain microvascular endothelium. Platelets were found to act as a bridge between CD36-deficient brain endothelium and IEs via CD36 binding. This mechanism possibly bestows IEs with more diversified binding properties, promoting CM pathogenesis [40].

PfEMP-1 variants expressed on the surface of IEs determine the cytoadherence properties of parasite populations [35]. These proteins are well-described ligands, and studies have shown that they recognize and interact with more than one receptor independently of IEs subpopulations. One PfEMP-1 variant may allow the cytoadherence to more than one host receptor through its semi-conserved N-terminal head structure DBL1α and CIDR1α [41]. Likewise, DBLβ2 and DBLβ3 bind ICAM-1 [42–44]. The tandem NTS-DBLα-CIDRγ of *varO* is essential for IEs binding and rosette formation [45]. CIDRα1 of DC8 and DC13 *var* genes is a potential ligand of endothelial protein C receptor [44]. Moreover, multiple extracellular domains of DC8 of IT4var19 bound brain and non brain endothelial cells [46].

In this study, the transcription of *pfemp-1* variants including groups A, B, *var3*, DC8, and DC13 was clearly up-regulated in CM parasites as compared to UM isolates, in agreement with previous studies [8–11], supporting the implication of these genes in CM pathogenesis. *var2csa* is transcribed to a much higher level in isolates from pregnant than from non-pregnant women [30]. Some CM isolates expressed *var2csa* at a higher level than in UM parasites. However, binding to CSPG was consistently very weak and similar in both UM and CM groups, indicating that VAR2CSA does not take part in malaria pathogenesis in children. Although VAR2CSA expression was absent at the surface of IEs in isolates from children in other studies, its transcription was reported to be high in these isolates [8, 47].

The transcription level of group B genes positively correlated with the binding level of IEs to CD36, confirming the earlier findings that parasite ligands for CD36 are PfEMP-1 variants encoded by *var* genes belonging to groups B and C through their CIDR1α domain [48, 49]. Functional analysis using mutagenesis in the M2 region of CIDR1α linked CD36 binding ability to specific CIDR1α residues [50]. Recombinant protein of CIDRα1.1 of DC8 also inhibits the binding of IEs to brain endothelium cells, suggesting a role of this domain in binding phenotypes involved in cerebral malaria. However, in this study, parasite lines that express IT4var6 and IT4var19 genes containing DC8 at high level and IT4var13 at low level were shown to bind weakly to CD36 [13]. Other studies using parasite lines selected to express only one *pfemp-1* variant are needed to demonstrate specific PFEMP-1/host receptor combinations.

The transcription levels of DC8 and DC13 were higher in CM isolates as compared to those in UM isolates, but were not correlated with the binding levels to CD36, ICAM-1, or CSPG. DC8 and DC13 are part of few genes from groups B and A, respectively. These two domain cassettes have been shown to mediate the binding of IEs to EPCR [51]. Positive correlation between DC8 and 13 expression and elevated binding to EPCR could be expected in CM parasites. It is possible that the present study did not include sufficient number of isolates expressing DC8 and DC13 and, for this reason, may have lacked the statistical power to detect a possible correlation between binding phenotypes and transcription patterns. On another hand, together with PfEMP1 or alone other parasite proteins expressed on the surface of IEs may take part in cytoadherence mechanism making difficult to reveal such correlation.

PfEMP-1 architecture is not randomly arranged since it has been observed that specific arrangement of CIDR and DBL domain tandems occurs more frequently to maintain a minimum structural function for PfEMP1 folding, export, and cytoadherence [52]. Therefore, the correlation among transcription patterns of group B variants, which are characterized by CIDR and DBL domains, binding phenotype to CD36, and severity of the disease reflects the existence of conserved phenotypes associated with cerebral malaria.

## Conclusion

A higher number of CM isolates binds more to CD36, as compared to UM isolates, but there was no significant difference between CM and UM isolates in the binding levels to ICAM-1 and CSPG. CM isolates transcribed groups A, B, *var2csa, var3*, DC8, and DC13 at higher levels than UM isolates. These results support the role of these PfEMP-1 variants in CM. Likewise, the positive correlation between the high transcription level of group B *var* genes and the increased binding of isolates from CM patients strengthens the implication of group B *var* encoding PfEMP-1 in the binding interaction with CD36 during cerebral malaria.

For its survival, the parasite has developed sophisticated mechanisms to bind to host receptors and establish acute or chronic infections. Hence, the identification of PfEMP-1 domains of group B var genes, probably CIDR1 domains, involved in CD36 binding during cerebral malaria could be targeted to prevent severe and complicated malaria.

## Abbreviations

CD36: Cluster of differentiation
CM: Cerebral malaria
CSA: Chondroitin sulphate A
CSPG: Chondroitin sulfate proteoglycan
DC: Domain cassettes
HBMEC: Human brain microvascular endothelial cells
ICAM-1: Intercellular adhesion molecule 1
IEs: *Plasmodium falciparum*-infected erythrocytes
PfEMP-1: *P. falciparum* Erythrocyte Membrane Protein-1
UM: Uncomplicated malaria.
